# LiDAR point cloud denoising for individual tree extraction based on the Noise4Denoise

**DOI:** 10.3389/fpls.2024.1490660

**Published:** 2025-01-09

**Authors:** Xiangfei Lu, Zongyu Ye, Liyong Fu, Huaiyi Wang, Kaiyu Wang, Yaquan Dou, Dongbo Xie, Xiaodi Zhao

**Affiliations:** ^1^ Research Institute of Forest Policy and Information, Chinese Academy of Forestry, Beijing, China; ^2^ School of Mechanical and Materials Engineering, North China University of Technology, Beijing, China; ^3^ Research Institute of Forest Resource Information Technique, Chinese Academy of Forestry, Beijing, China; ^4^ Information Technology, Belarusian State University, Minsk, Belarus

**Keywords:** individual tree extraction, LiDAR point clouds, point cloud denoising, deep learning, ecological study

## Abstract

The processing of LiDAR point cloud data is of critical importance in the context of forest resource surveys, as well as representing a pivotal element in the realm of forest physiological and ecological studies.Nonetheless, conventional denoising algorithms frequently exhibit deficiencies with regard to adaptability and denoising efficacy, particularly when employed in relation to disparate datasets.To address these issues, this study introduces DEN4, an unsupervised, deep learning-based point cloud denoising algorithm designed to improve the accuracy of single tree segmentation in LiDAR point clouds.DEN4 introduces a multilevel noise separation module that effectively distinguishes between signal and noise, thereby improving the signal-to-noise ratio (SNR) and reducing the error.The experimental results demonstrate that DEN4 significantly outperforms traditional denoising methods in several key metrics, including mean square error (MSE), SNR, Hausdorff distance, and structural similarity index (SSIM).In the 60 sample dataset, DEN4 achieved the best mean and standard deviation on all metrics: Specifically, the MSE mean was found to be 0.0094, with a standard deviation of 0.0008, the SNR mean was 149.1570, with a standard deviation of 0.5628, the Hausdorff mean was 0.8503, with a standard deviation of 0.0947, and the SSIM mean was 0.8399, with a standard deviation of 0.0054. For instance, in the S10 dataset, DEN4 attained a 70.2% diminution in MSE and a 37.8% augmentation in SNR in comparison with PTD.The findings demonstrate the efficacy of DEN4 in multiple forest datasets, its ability to maintain geometric integrity, and its enhanced stability without the necessity for pre-labelled data. The algorithm's superior performance and robustness in diverse forest environments underscores its potential application in single tree segmentation and forest resource management.

## 1 Introduction

Forests constitute a crucial component of the Earth’s ecosystem, playing an indispensable role in maintaining ecological balance and supporting a myriad of life forms (Menut et al., 2023; Sun et al., 2023). They provide an extensive array of ecosystem services, which include but are not limited to, biodiversity conservation, carbon storage, and the preservation of cultural values (Sabatini et al., 2018; Handegard et al., 2024). Firstly, it is estimated that forest ecosystems contain more than half of the world’s biodiversity and include species of significant conservation value (Myers et al., 2000; Lewis et al., 2015). Secondly, while conserving energy and reducing carbon dioxide emissions, exploring the carbon sequestration potential of forest ecosystems is key to achieving carbon neutrality (Piao et al., 2022), as forest ecosystems absorb nearly one-third of the anthropogenic carbon dioxide emissions each year (Harris et al., 2021). Additionally, beyond providing food, maintaining soil, and ensuring climate stability (Millennium ecosystem assessment, 2005), forest ecosystems can have a positive impact on human physical and mental health through individual engagement (Daniel et al., 2012), thereby offering psychological and cultural benefits to both individuals and society (Ko and Son, 2018). In the future development of humanity, forest ecosystems play an extremely important role, making the study of forest physiology and ecology, as well as the management of forests, indispensable (Jandl et al., 2007; Cox et al., 2023).

Light Detection and Ranging(LIDAR) plays a significant role in forest physiology and ecology research, providing valuable data for forest surveys and management (Yong Pang et al., 2005). Airborne LIDAR can accurately extract canopy vertical structures, allowing the acquisition of forest parameters at both the stand and individual tree scales (Li et al., 2016) Unmanned Aerial Vehicle(UAV)-mounted LIDAR can control scanning errors to within centimeters or even millimeters, offering significant advantages in digital ecosystem construction, aboveground biomass estimation, and long-term monitoring of stand environments (Guo et al., 2014; Liu et al., 2017). It has become a consensus among global forestry scholars that using UAV-mounted LIDAR to obtain forest ecological data is essential for strengthening forest ecosystem management. Currently, image segmentation based on the Canopy Height Model (CHM) and point cloud segmentation based on normalized point cloud spatial clustering are two typical methods for acquiring forest ecological data using UAV-mounted LIDAR (Xu et al; Cao et al., 2013; Li et al., 2016). Point cloud segmentation based on UAV-mounted LIDAR data is crucial for addressing various issues such as measuring canopy height, identifying individual trees, estimating leaf area index and canopy closure, and calculating biomass (Maltamo et al., 2004; Riaño et al., 2004; Zhao et al., 2009; Liu and Pang, 2014).

However, due to the limitations of the scanning equipment’s precision and the influence of the acquisition environment, point cloud data inevitably contains many noise points and outliers (Lu and zou, 2019). The presence of these noise points reduces the accuracy of the data, severely impacting the subsequent data processing and usage. To reduce noise in point cloud data and obtain cleaner point clouds, many scholars have conducted in-depth research and exploration. Currently, the most widely used and mature technology for denoising is filtering-based techniques. Choudhury et al. proposed a trilateral filtering method, building on the bilateral filtering approach, for denoising 3D mesh models (Choudhury and Tumblin, 2003). Schall et al. confined the filtering window to the approximate normal vector region for normal vector filtering (Schall et al., 2008). On the basis of the SUN algorithm, Xinhe Liang et al. filtered both the normal vectors of the discrete point model and the positions of the points to develop an improved discrete point model filtering method (Liang et al., 2010). Dongdong Lu et al. explored the application of statistical filtering algorithms and radius filtering algorithms in practical denoising tasks and conducted a comparative analysis of the advantages and disadvantages of these two typical filters in terms of denoising performance (Lu and zou, 2019). In the field of LIDAR-based individual tree extraction, filtering methods are also widely used. For example, Yushan Guo et al. employed morphological filtering methods in their study on individual tree crown extraction to ensure that contour information is well preserved (Guo et al., 2016). Xiaokang Wu compared multi-level surface filtering algorithms, slope filtering algorithms, and CSF filtering algorithms, with results showing that in the field of LIDAR-based individual tree extraction, CSF filtering offers higher accuracy than slope filtering and multi-level surface filtering methods (Wu et al., 2023). Additionally, Kaisen Ma, Wei Li, and others processed LIDAR point cloud data using an improved progressive triangulated network filtering method (Ma, 2023; Li and Wang, 2024). However, traditional algorithms have certain limitations in adaptability and denoising effectiveness, particularly when handling different types of data where the performance can vary significantly.

With the development of various technologies, point cloud denoising based on deep learning and machine learning has gradually attracted the attention of many scholars. Duan Chaojing et al. proposed a neural network-based 3D point cloud denoising framework that can accurately estimate and remove noise in point clouds (Duan et al., 2019). Jie Zhang et al. introduced an unsupervised deep point cloud denoising algorithm guided by density priors, which improves the performance of unsupervised deep point cloud denoising algorithms (Jie et al., 2024). Luo Shitong and others proposed a neural network architecture designed to estimate relevant scores using only noisy point clouds as input and developed a denoising algorithm based on the estimated scores (Luo and Hu, 2021). Pedro Hermosilla Casajus, Tobias Ritschel, and Timo Ropinski proposed an unsupervised point cloud denoising method that extends unsupervised image denoising techniques to the 3D point cloud domain, introducing spatial priors to guide point clouds toward the real manifold (Casajus et al., 2019).

Currently, point cloud denoising technology is still at a stage where deep learning methods and traditional methods are developing in parallel. However, compared to traditional algorithms, deep learning methods, by utilizing advanced architectures such as Convolutional Neural Networks (CNN) and Graph Convolutional Networks (GCN), can automatically learn and extract complex features from point clouds, significantly reducing the workload of manual feature design. Through training on large datasets, deep learning models can effectively adapt to different types of noise and variations in point cloud data. For example, the model can learn specific noise patterns from the training data, enabling it to perform denoising more effectively. Additionally, deep learning methods excel in handling complex scenarios and diverse point cloud data, particularly in challenging situations involving non-uniform distributions, high-dimensional data, and multi-view point cloud fusion (Menut et al., 2023). These methods not only enhance denoising effectiveness but also preserve more details and structural information in the point cloud (Mao et al., 2022). However, in the field of LIDAR-based individual tree extraction, traditional algorithms still dominate, and deep learning methods have not yet fully replaced traditional methods when addressing more detailed and complex data requirements. This indicates that there is still room for further optimization and development of deep learning methods in individual tree extraction tasks.

This paper aims to develop an unsupervised deep learning algorithm for LIDAR-based individual tree extraction, building on existing deep learning algorithms. The specific objectives include: collecting and preprocessing LIDAR point cloud data for individual tree extraction; selecting suitable algorithms for unsupervised learning, and improving and optimizing their design; training and optimizing the model to enhance denoising effectiveness; conducting detailed quantitative and qualitative analyses of the denoising results; and optimizing the denoising process to lay a foundation for future data processing and applications, ensuring that the data can be more effectively utilized in subsequent analyses and research.

## 2 Materials and methodology

### 2.1 Dataset and data preprocessing

**Figure 1:**
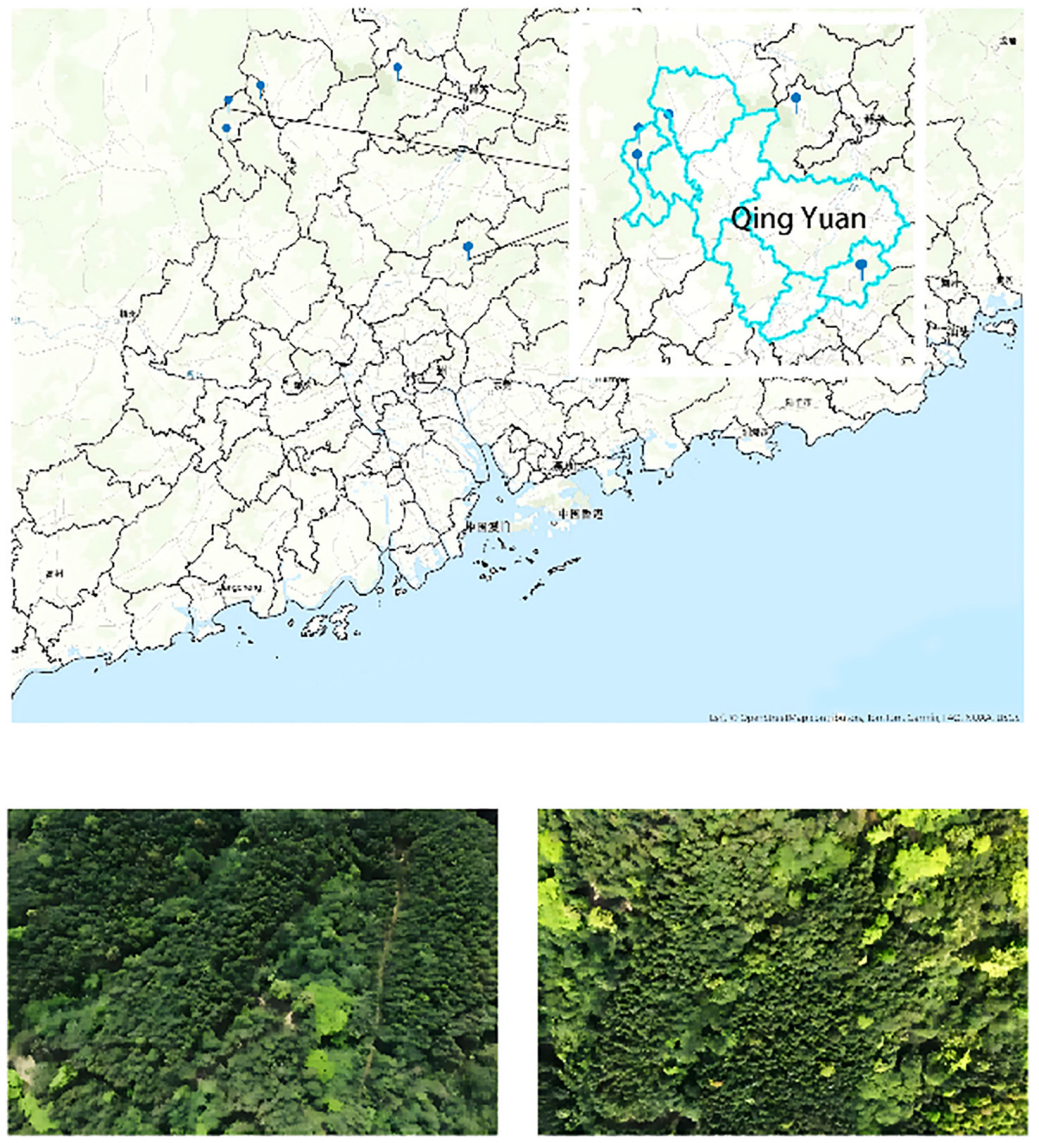
Schematic of the plot.

The data used in this study were collected from 17 forest plots located in Qingyuan City, Guangdong Province. The actual sampling plots cover an area of 80*80 meters, and this data was collected through UAV flight scans following manual ground surveys. The data was collected in March 2024 using a BB4 UAV equipped with an AS-1300HL Lidar system. The laser scanner model used was the Riegl VUX-1LR, with a wavelength of 1550 nm, a pulse length of 3.5 ns, and a laser beam divergence angle of 0.5 mrad. The pulse repetition frequency was 50 kHz, with a maximum scanning angle of 30°, and a scanning frequency of 49 Hz. The UAV followed a grid flight pattern, with a point cloud overlap rate of 50% and an average flight speed of 10 m/s. The average point cloud density for the plots was 110 pts/m². A schematic of the plot is shown in **Figure 1**.

The dataset comprises 17 forest plots in Qingyuan City, Guangdong Province, selected for their ecological complexity and biodiversity. Guangdong’s forests, spanning tropical and subtropical climates, present diverse vegetation and challenging noise conditions, providing a robust test for the algorithm. Compared to some public datasets and datasets provided by other research studies, the dataset used in this paper has undergone an actual manual ground survey and includes a large amount of field imagery. These field images can be directly correlated with the point cloud data, allowing for adjustments and comparisons based on the images during later processing, thereby improving the accuracy and precision of the data handling.

Before data cleaning, the collected data underwent preliminary processing and classification in this study. The point cloud data was matched with actual imagery to ensure consistency between the point cloud data and the real geographic location. During the processing, specific functions were used to handle the point cloud data, generating arrays containing the point cloud data and storing them in the required format for use in subsequent steps. After segmenting the plot data, 60 samples were selected as experimental subjects.

To improve the stability of model training, accelerate model convergence, reduce bias, and facilitate the comparison of various algorithms in later stages, the point cloud data was also normalized. This step ensured the comparability of the data across different algorithms and models, enhancing the reliability and applicability of the research results.

### 2.2 Foundations of the algorithm

This study is designed based on the principles of the Noise4Denoise(DEN4) (Wang et al., 2024) algorithm. The algorithm is redesigned and its steps are organized accordingly to meet the requirements of Lidar-based individual tree extraction and point cloud denoising. The decision to choose the Noise4Denoise algorithm in this study is primarily based on its proven effectiveness and well-documented experimental results. Additionally, the algorithm’s classification as an unsupervised learning method offers significant advantages. Unsupervised algorithms do not require labeled training data, allowing them to learn directly from unlabeled datasets. This capability makes them particularly well-suited for large-scale, complex datasets, especially in situations where labeling is costly or impractical. Furthermore, unsupervised algorithms are highly versatile, performing effectively across various environments, regardless of whether the data is high-dimensional, unstructured, or lacks clear classifications or labels. This broad applicability underlines their potential in a wide range of data analysis tasks.

The training process of the algorithm is based on the following fundamental relationships. Consider a clean point cloud ***P*** with nnn points, where the three-dimensional coordinates of the points are given as


(1) $$\begin{array}{c} \text{P}=\left\{{\text{p}}_{\text{i}}│\text{i}=1,2,\ldots ,\text{n}\right\} \end{array}$$

where $\;{\text{p}}_{\text{i}}\in {\text{R}}^{3}\;$ represents the coordinates of a point in three-dimensional space. Then, a point cloud with noise can be defined as


(2) $$\begin{array}{c} \dot{\text{P}}=\left\{{\dot{\text{p}}}_{\text{i}}│\text{i}=1,2,\ldots ,\text{n}\right\}=\text{P}+\text{N} \end{array}$$

where ***N*** represents the noise. A new noise ***M*** is redefined, with the same resampling range as ***N***, but ***M*** and ***N*** are independent and not identical. Adding ***N*** to $\dot{\text{P}}$ yields another point cloud P˙'


(3) P˙'={p˙i'│i=1,2,…,n}=P˙+M=P+M+N

Here, P˙'  contains double the noise, encompassing noise from both ***N*** and ***M***. The algorithm assumes that the clean point cloud can be estimated based on the input noise. P˙' is set as the prior value, representing the observable point cloud with double noise. The estimation of the overall surface of the noisy point cloud is expressed by the expectation E(P˙│P˙') .According to Equation 2, we have


(4) E(P˙│P˙')=E(P+N│P˙')=E(P│P˙')+E(N│P˙')

Since ***M*** and ***N*** are independently and identically distributed, and both are sampled from the same distribution range, their expected values are the same. That is E(M│P˙')=E(N│P˙') . Therefore, it can be deduced that:


(5) 2E(P˙│P˙')= E(P│P˙')+E(P˙'│P˙')

And then, it can be deduced as


E(P│P˙')= 2E(P˙│P˙')−E(P˙'│P˙')

(6) E(P│P˙')−E(P˙'│P˙')= E(P˙│P˙')−E(P˙'│P˙')

Here, using E(P│P˙')=P¯ 、 E(P˙│P˙')=P˙¯ 、 E(P˙'│P˙')=P'˙¯ , P¯、P˙¯、P'˙¯  represent the predicted values of $\text{P}、\dot{\text{P}}$ and ${\dot{\text{P}}}^{'}$ respectively, substituting these into Equation 6, we can obtain:


(7) $$\begin{array}{c} \bar{\text{P}}-\bar{\dot{\text{P}}}=\bar{\dot{\text{P}}}-\bar{\dot{{\text{P}}^{\prime }}} \end{array}$$

According to the rules of conditional expectation and the discrete nature of the point cloud, it can be derived that P'˙¯= E(P˙'│P˙') . Assuming that the values in  P¯、P˙¯、P'˙¯ correspond one-to-one, a noise displacement vector can be predicted for each point in P˙' ′, and these vectors can be used to construct an approximate clean point cloud $\bar{P}\;$ . This relationship can be expressed as


(8) $$\begin{array}{c} \bar{\text{P}}=\left\{{\bar{\text{p}}}_{\text{i}}│\text{i}=1,2,\ldots ,\text{n}\right\}=\left\{{\dot{\text{p}}}_{\text{i}}^{'}+\bar{{\text{d}}_{i}^{'}}│\text{i}=1,2,\ldots ,\text{n}\right\} \end{array}$$

where $\bar{{\text{d}}_{i}^{'}}$ represents the predicted displacement vector for the point $\;{\dot{\text{p}}}_{\text{i}}^{'}$ ; based on these vectors, the corresponding positions of the clean point cloud can be directly estimated.

Adding another set of displacement vectors to ${\dot{P}}^{'}$ yields the predicted value $\bar{\dot{P}}$ , which can be expressed as


(9) $$\begin{array}{c} \bar{\dot{\text{P}}}=\left\{{\bar{\dot{\text{p}}}}_{\text{i}}│\text{i}=1,2,\ldots ,\text{n}\right\}=\left\{{\dot{\text{p}}}_{\text{i}}^{'}+\bar{{\text{d}}_{i}^{'}}│\text{i}=1,2,\ldots ,\text{n}\right\} \end{array}$$

Based on the one-to-one correspondence assumption, by substituting the values into Equation 7, it can ultimately be proven that the direct prediction $\bar{d_{i}^{'}}$ is equivalent to twice ${\bar{d}}_{i}$ ​:


$$\bar{\text{p}}-\bar{\dot{\text{p}}}=\bar{\dot{\text{p}}}-{\dot{\text{p}}}_{\text{i}}^{'}$$

$$\left({\dot{\text{p}}}_{\text{i}}^{'}+\bar{{\text{d}}_{i}^{'}}\right)-\left({\dot{\text{p}}}_{\text{i}}^{'}+{\bar{\text{d}}}_{\text{i}}\right)=\left({\dot{\text{p}}}_{\text{i}}^{'}+{\bar{\text{d}}}_{\text{i}}\right)-{\dot{\text{p}}}_{\text{i}}^{'}$$

$$\bar{{\text{d}}_{i}^{'}}-{\bar{\text{d}}}_{\text{i}}={\bar{\text{d}}}_{\text{i}}$$

(10) $$\begin{array}{c} \bar{{\text{d}}_{i}^{'}}=2{\bar{\text{d}}}_{i} \end{array}$$

### 2.3 Model architecture

Noise4Denoise proposes an unsupervised point cloud denoising method, with the core idea of using a deep neural network to learn the mapping from noisy point clouds to clean point clouds. The specific implementation involves learning a displacement vector field that enables each noisy point cloud point to move to its predicted clean position. This process is achieved by training the network to minimize the difference between the denoised point cloud and certain criteria. The model is primarily composed of the following modules: Input Layer: The input consists of noisy point cloud data, typically in the form of double noise (i.e., adding two different noise patterns to the original point cloud). Feature Extraction Module: A deep learning-based network is used to extract both local and global features of the point cloud. Displacement Vector Prediction Module: Based on the extracted features, the model learns to predict a displacement vector for each point, enabling the mapping from noisy points to clean points. Output Layer: The output is the denoised point cloud, obtained by applying displacement corrections to the noisy points.

The design of the feature extraction module draws on network architectures such as PointNet and DGCNN, learning both local and global features to capture the geometric structure of the point cloud. To ensure the retention of point cloud details during denoising, the feature extraction module includes local neighborhood feature extraction. This is achieved by determining the neighborhood of each point using the k-Nearest Neighbors (k-NN) method, followed by extracting local geometric features through graph convolution. Global feature aggregation is achieved through global pooling, which integrates local features into a global descriptor.

The displacement vector prediction module receives the features output by the feature extraction module and generates a displacement vector for each point. To preserve the structure and details of the point cloud, the model is designed to use an MLP network to perform nonlinear transformations on the extracted features to generate the displacement vectors. A residual connection is added between the generated displacement vectors and the original point coordinates to ensure the stability and expressive power of the model.

**Figure 2:**
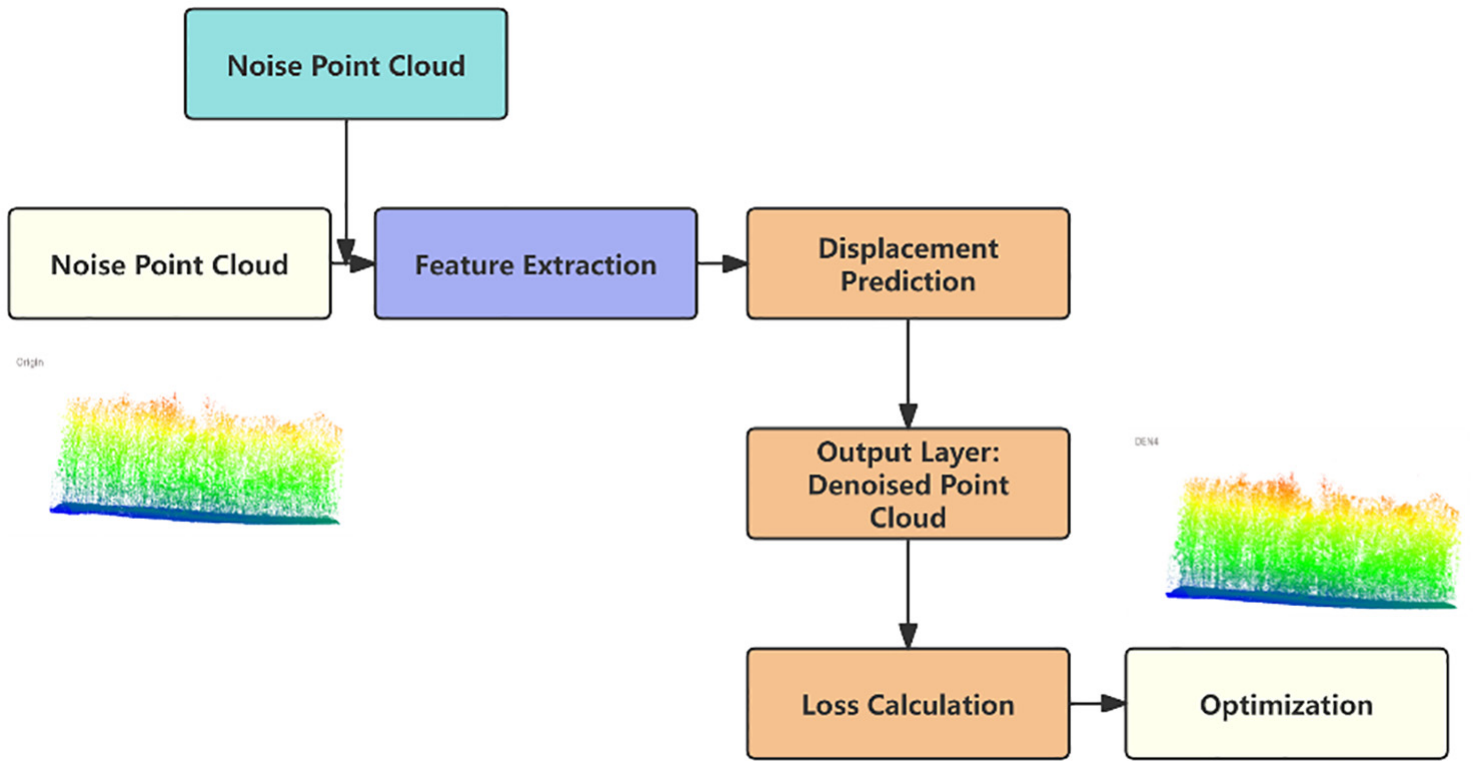
Model architecture.

To preserve the structure and details of the point cloud during the denoising process, the model incorporates a specifically designed symmetric loss function, ensuring that the overall geometric shape of the point cloud is not disrupted; Through local feature extraction and the relationship between neighboring points, the model can identify and preserve local structures; Regularization techniques such as Dropout or weight decay are employed to prevent the model from overfitting, thereby better preserving the details of the original point cloud. Our specific model architecture is shown in **Figure 2**.

### 2.4 Loss function

Using $\;\dot{{\text{d}}_{\text{i}}}={\dot{\text{p}}}_{\text{i}}-{\dot{\text{p}}}_{\text{i}}^{'}$ , the prediction of the actual value of ${\bar{\text{d}}}_{\text{i}}$ in Equation 10 guides the estimation of the displacement d'¯i . A mean squared error (MSE) loss function is designed, which can be defined as


(12) $$\begin{array}{c} {\text{L}}_{\text{mse}}=\|\bar{{\text{d}}_{i}^{'}}-2{\dot{{\text{d}}_{\text{i}}}\|}_{2}^{2} \end{array}$$

To ensure that the point cloud spacing after denoising remains appropriate and to avoid potential clustering issues, the algorithm incorporates a repulsion loss based on references (Rakotosaona et al., 2019; Zhang et al., 2020). This repulsion loss is designed to assist in the distribution of the clean point cloud. First, a pseudo-clean point cloud $\tilde{P}\;$ is defined as


(13) $$\begin{array}{c} \tilde{\text{P}}=\left\{{\dot{\text{p}}}_{\text{i}}^{'}+2\dot{{\text{d}}_{\text{i}}}│\text{i}=1,2,\ldots ,\text{n}\right\} \end{array}$$

where $\tilde{P}\;$ does not actually exhibit a clean appearance. The following process is used to define the pseudo-clean point cloud:


(14) $$\begin{array}{c} {\tilde{\text{P}}}_{\text{i}}=\left\{{\tilde{\text{p}}}_{\text{j}}│\|{\tilde{\text{p}}}_{\text{j}}-{\dot{\text{p}}}_{\text{i}}{\|}_{2,}{\tilde{\text{p}}}_{\text{j}}\in \tilde{\text{P}}\right\} \end{array}$$

Here, since ${\dot{\text{p}}}_{\text{i}}$ ​ is assumed to be a point cloud less affected by noise, ${\tilde{\text{p}}}_{\text{j}}\in \tilde{\text{P}}$ is used to query ${\tilde{\text{P}}}_{\text{i}}$ ​. Then, the point set ${\tilde{\text{P}}}_{\text{i}}$ ​ is normalized. Using these, the loss function can be defined as


(15) $$\begin{array}{c} {\text{L}}_{\text{rep}}=\underset{{\tilde{\text{p}}}_{\text{j}}\in {\tilde{\text{P}}}_{\text{i}}}{\text{max}}\|({\dot{\text{p}}}_{\text{i}}^{'}+\bar{{\text{d}}_{i}^{'}})-{\tilde{\text{p}}}_{\text{j}}{\|}_{2}^{2} \end{array}$$

Then the loss function ***L*** can be defined as


(16) $$\begin{array}{c} \text{L}={\text{L}}_{\text{mse}}+\text{γ}{\text{L}}_{\text{rep}} \end{array}$$

where γ  is a factor controlling the influence of the repulsion loss, set to 0.0005.

## 3 Results

### 3.1 Experimental conditions

#### 3.1.1 Hardware environment

The hardware configuration used in this study is as follows: a computer equipped with an Intel(R) Core(TM) i5-10500 processor, with a base frequency of 3.1GHz, featuring 6 cores and 12 threads; a NVIDIA GeForce RTX 2070 SUPER graphics card with 8GB of VRAM; the computing platform is also equipped with 32GB of RAM and 4TB of storage space to support data processing and experimental computations.

#### 3.1.2 Software environment

This paper describes the computational experiments conducted under the Windows 11 Pro operating system, utilizing CUDA 12.5 as the parallel computing framework. To facilitate model training management, PyCharm was used as the development environment, and CloudCompare was employed for the visualization of point cloud data.

### 3.2 Hyperparameter configuration

In this experiment, the settings for various parameters were determined through exploratory experiments. For the learning rate, a cosine annealing algorithm was used to make an initial estimate, with a range from 0.1 to 0.0001, and 0.001 was ultimately selected as the optimal learning rate. Additionally, the weight parameter in the loss function was set to 0.0005, following the original algorithm. For the noise addition level, after testing different noise intensities ranging from 0.01 to 0.1, 0.1 was chosen as the optimal value. The structure of the encoder and decoder was optimized using a layer-by-layer approach, resulting in a 3-256-512-1024-2048 architecture, which struck a good balance between experimental results and computation time. A total of 200 training epochs were conducted, with each training batch containing 128 samples.

### 3.3 Common evaluation metrics for point cloud denoising

The commonly used evaluation methods for Lidar point cloud denoising include Mean Squared Error (MSE), Signal-to-Noise Ratio (SNR) (Mao et al., 2022), Hausdorff Distance (Javaheri et al., 2020), and Structural Similarity Index (SSIM) (Liu et al., 2022). This study selects MSE, SNR, Hausdorff Distance, and SSIM as evaluation criteria because they comprehensively assess the effectiveness of point cloud denoising from different dimensions. MSE evaluates the accuracy of the algorithm, SNR measures the clarity of the signal, Hausdorff Distance assesses geometric precision, and SSIM focuses on structural fidelity. By integrating these four metrics, it is possible to evaluate the performance of denoising algorithms holistically and accurately, thereby providing a better understanding of the algorithm’s effectiveness and limitations in practical applications.

(1) MSE refers to the expected value of the squared difference between the estimated parameter and the true parameter value. It is used to describe the extent of the difference in the point cloud before and after denoising. MSE quantifies the prediction error of the algorithm or the accuracy of the model by calculating the average of the sum of the squared errors between the predicted values and the actual values. The formula can be expressed as


(17) $$\begin{array}{c} \text{m}s\text{e}=\frac{1}{\text{m}}\sum_{\text{i}=1}^{\text{m}}{\left({\text{y}}_{\text{i}}-{\hat{\text{y}}}_{\text{i}}\right)}^{2} \end{array}$$

where ${\text{y}}_{\text{i}}\;$ represents the true value, and ${\hat{\text{y}}}_{i}$ ​ represents the predicted value. The smaller the MSE, the closer the model’s prediction is to the true value. Therefore, in the evaluation of point cloud denoising, a lower MSE indicates better denoising performance.

(2) SNR is used to measure the ratio of the power of a signal to the power of the noise, typically expressed in decibels (dB). In point cloud processing, SNR describes the relative strength of the signal to the noise in the point cloud, reflecting the clarity of the signal and the degree of noise interference. The ratio of the signal power to the noise power is commonly multiplied by 10 in its logarithmic form, and the specific formula is as follows:


(18) $$\begin{array}{c} \text{SNR}=10\text{lg}\frac{{\text{P}}_{\text{s}}}{{\text{P}}_{\text{n}}} \end{array}$$

where ${\text{P}}_{\text{s}}$ ​ represents the power of the signal, and $\;{\text{P}}_{\text{n}}$ ​ represents the power of the noise. The logarithmic operation in the formula amplifies the difference between the signal and noise power, allowing SNR to intuitively express the quality of the signal in decibels.

(3) Hausdorff Distance is a metric used to describe the degree of similarity between two sets of points, measuring the maximum distance between two point clouds. It is often used to assess the geometric differences between point clouds before and after denoising. Specifically, the Hausdorff distance is defined as the maximum distance from a point in one set to the nearest point in another set. If we consider two point sets AAA and BBB, the one-way Hausdorff distance between these two sets can be expressed as


(19) $$\begin{array}{c} \text{h}\left(\text{P},\dot{\text{P}}\right)=\underset{{\text{p}}_{\text{i}}\in \text{P}}{\text{max}}\underset{{\dot{\text{p}}}_{\text{i}}\in \text{P}}{\text{min}}\|{\text{p}}_{\text{i}}-{\dot{\text{p}}}_{\text{i}}\| \end{array}$$

where $p_{i}-{\dot{p}}_{i}$ represents the Euclidean distance between $p_{i}$ ​ and ${\dot{\text{p}}}_{i}$ ​, and $\text{h}\left(\text{P},\dot{\text{P}}\right)$ is also known as the forward Hausdorff distance (Tie, 2024). The Hausdorff distance emphasizes the farthest minimum distance between two point sets, making it sensitive to capturing the maximum differences between point clouds. This makes it a powerful tool for evaluating point cloud denoising effectiveness, especially in applications where maintaining geometric accuracy is crucial.

(4) SSIM is an important metric for measuring the similarity between two images, particularly useful for capturing changes in structural information. It is also widely used to assess the structural similarity between two point clouds. In 3D point cloud processing, SSIM can be calculated by projecting the 3D point cloud onto a 2D plane, or by using a voxelization method to segment the point cloud for calculation. In this study, we adopt a neighborhood-based approach to directly compute SSIM in 3D space. The basic formula for SSIM is as follows:


(20) $$\begin{array}{c} \text{SSIM}\left(x,y\right)=\frac{\left(2{\text{μ}}_{\text{x}}{\text{μ}}_{\text{y}}+{\text{C}}_{1}\right)\left(2{\text{σ}}_{\text{xy}}+C_{2}\right)}{\left({\text{μ}}_{x}^{2}+{\text{μ}}_{y}^{2}+{\text{C}}_{1}\right)\left({\text{σ}}_{x}^{2}+{\text{σ}}_{y}^{2}+{\text{C}}_{2}\right)} \end{array}$$

where ${\text{μ}}_{\text{x}}$ ​ and ${\text{μ}}_{\text{y}}$ ​ are the mean values of images ***x*** and ***y*** respectively; ${\text{σ}}_{x}^{2}$ and ${\text{σ}}_{y}^{2}$ are the variances of ***x*** and ***y*** respectively; and $\sigma_{xy}$ is the covariance between images ***x*** and ***y***. Where ${\text{C}}_{1}={\left({\text{K}}_{1}\text{L}\right)}^{2}$ and ${\text{C}}_{2}={\left({\text{K}}_{2}\text{L}\right)}^{2}$ are two constants added to avoid division by a small denominator, with ***L*** representing the dynamic range of the pixel values. In the application of 3D point clouds, SSIM can be used to measure the structural similarity of point clouds before and after denoising by analyzing both the local structure and global distribution of the point cloud. This method allows for a more effective evaluation of the point cloud processing algorithm’s ability to preserve geometric structure and details.

### 3.4 Results analysis

#### 3.4.1 Quantitative analysis

This paper analyzes the experimental results of 60 treated sample plots and additionally presents the point cloud data processing results of two specific plots, named S5 and S10. To evaluate the accuracy, generality, and effectiveness of the algorithm used in Lidar point cloud processing for individual tree extraction, this paper compares several common denoising methods, including Morphological filtering, Progressive Triangulated Network Denoising(PTD), Statistical Outlier Removal(SOR), and Radius Outlier Removal(ROR). These methods encompass a range of processing dimensions, allowing for a more comprehensive comparison that highlights the adaptability of the deep learning algorithm in handling diverse data characteristics. By comparing these traditional methods, it becomes possible to evaluate the robustness and adaptability of the deep learning algorithm across various denoising scenarios. This comparative analysis not only showcases the innovation and efficacy of the deep learning algorithm in point cloud denoising but also clarifies its advantages over traditional methods in practical applications. Moreover, this analysis provides valuable insights into the algorithm’s performance, offering essential guidance for its further optimization and real-world implementation.

Plots S5 and S10 were chosen for analysis due to their complexity, which makes them highly representative. Firstly, these plots encompass both densely forested areas and relatively sparse regions, capturing a diverse range of forest conditions. Secondly, their rugged terrain further highlights their suitability as representative samples for this study.

To quantify the denoising performance of these methods, this paper calculates the Mean Squared Error (MSE), Signal-to-Noise Ratio (SNR), Hausdorff Distance, and Structural Similarity Index (SSIM), and compares the results across several algorithms. MSE and SNR provide distinct yet complementary measures of denoising quality. Combining these two metrics allows for a more comprehensive evaluation of algorithm performance. SSIM and Hausdorff Distance, on the other hand, assess the denoising process from different perspectives—structural fidelity and geometric accuracy, respectively. By comparing SSIM and Hausdorff Distance together, we can evaluate the performance of denoising algorithms in terms of both structural similarity (measured by SSIM) and geometric precision (measured by Hausdorff Distance). This dual analysis offers a more thorough assessment of algorithm effectiveness, ensuring that the algorithm not only preserves the visual and structural similarity of the point clouds but also retains their precise geometric structure.

This study calculates the standard deviation and mean absolute deviation for various algorithms across four key metrics, with the results summarized in **Table 1**. Additionally, the outcomes for plots S5 and S10 are discussed in detail, as shown in **Tables 2**, **3**, respectively. In **Table 1**, the first metric analyzed is MSE. The algorithm DEN4 achieves the lowest mean MSE (0.0094) and the smallest standard deviation (0.0008), indicating the least error and highest stability. In contrast, other methods, such as Morphological and SOR, have mean MSE values ranging from 0.0297 to 0.0300, which are significantly higher than DEN4.The second metric is SNR. DEN4 exhibits the highest mean SNR (149.1570), reflecting the best signal quality post-denoising. Its smallest standard deviation (0.5628) further highlights its consistent performance in this regard. For the Hausdorff distance, DEN4 achieves the lowest mean value (0.8503), demonstrating superior preservation of the point cloud’s geometric structure. Its standard deviation is also the smallest (0.0947), underscoring its stability in maintaining geometric characteristics. Finally, in terms of SSIM, DEN4 delivers the highest mean value (0.8399) and the smallest standard deviation (0.0054), showcasing its outstanding structural similarity performance with minimal variability.

**Table 1:** Overall noise reduction sample standard deviation and absolute mean deviation.

Methodology	MSE_mean	MSE_std	SNR_mean	SNR_std	Hausdorff_mean	Hausdorff_std	SSIM_mean	SSIM_std
DEN4	0.0094	0.0008	149.1570	0.5628	0.8503	0.0947	0.8399	0.0054
Morphological	0.0299	0.0058	146.1743	1.1729	5.3568	1.2884	0.7649	0.0297
PTD	0.0297	0.0056	146.1301	1.1254	5.5593	1.4601	0.7752	0.0306
ROR	0.0301	0.0054	146.1726	1.0242	5.2390	1.4777	0.7727	0.0291
SOR	0.0300	0.0055	145.8800	1.0088	5.3034	1.3772	0.7724	0.0307

**Table 2:** MSE, SNR, Hausdorff and SSIM after S5 treatment Indicator calculation results.

Methodology	MSE	SNR	Hausdorff	SSIM
Morphological	0.0173	147.2195	7.5856	0.834
PTD	0.0296	144.9017	7.7872	0.726
SOR	0.021	146.3831	7.5609	0.8348
ROR	0.0198	147.5695	7.5782	0.834
DEN4	0.0106	149.3518	0.8116	0.7749

**Table 3:** MSE, SNR, Hausdorff and SSIM after S5 treatment Indicator calculation results.

Methodology	MSE	SNR	Hausdorff	SSIM
Morphological	0.017	147.3146	7.5856	0.834
PTD	0.0353	144.1307	7.7872	0.726
SOR	0.0222	146.1539	7.5609	0.8348
ROR	0.0169	147.3146	7.5782	0.834
DEN4	0.0105	149.4042	0.8116	0.7749

Specifically, in the S10 point cloud, the algorithm’s MSE value decreased by 70.2% compared to PTD and by 37.8% compared to ROR, reaching 0.0105. This indicates that the algorithm effectively maintains the accuracy of the point cloud data while reducing noise. The algorithm’s SNR value is 1-5 dB higher than other algorithms, reaching a maximum of 149.4042, indicating a significant advantage in enhancing signal quality. Additionally, the algorithm’s Hausdorff distance is significantly lower than other methods, at 0.8466, demonstrating its high precision in preserving the geometric structure of the point cloud. The algorithm’s SSIM value is slightly lower than that of Morphological Filtering and SOR, but the differences are minimal. Moreover, the algorithm exhibits a high degree of consistency in processing point cloud data across different plots (S5 and S10) and demonstrates good robustness. The MSE variation is 0.0001, SNR variation is 0.0523, Hausdorff variation is 0.0350, and SSIM variation is 0.130, with overall error within the range of 0% to 5%. Compared to other algorithms, it shows excellent stability.

**Figure 3:**
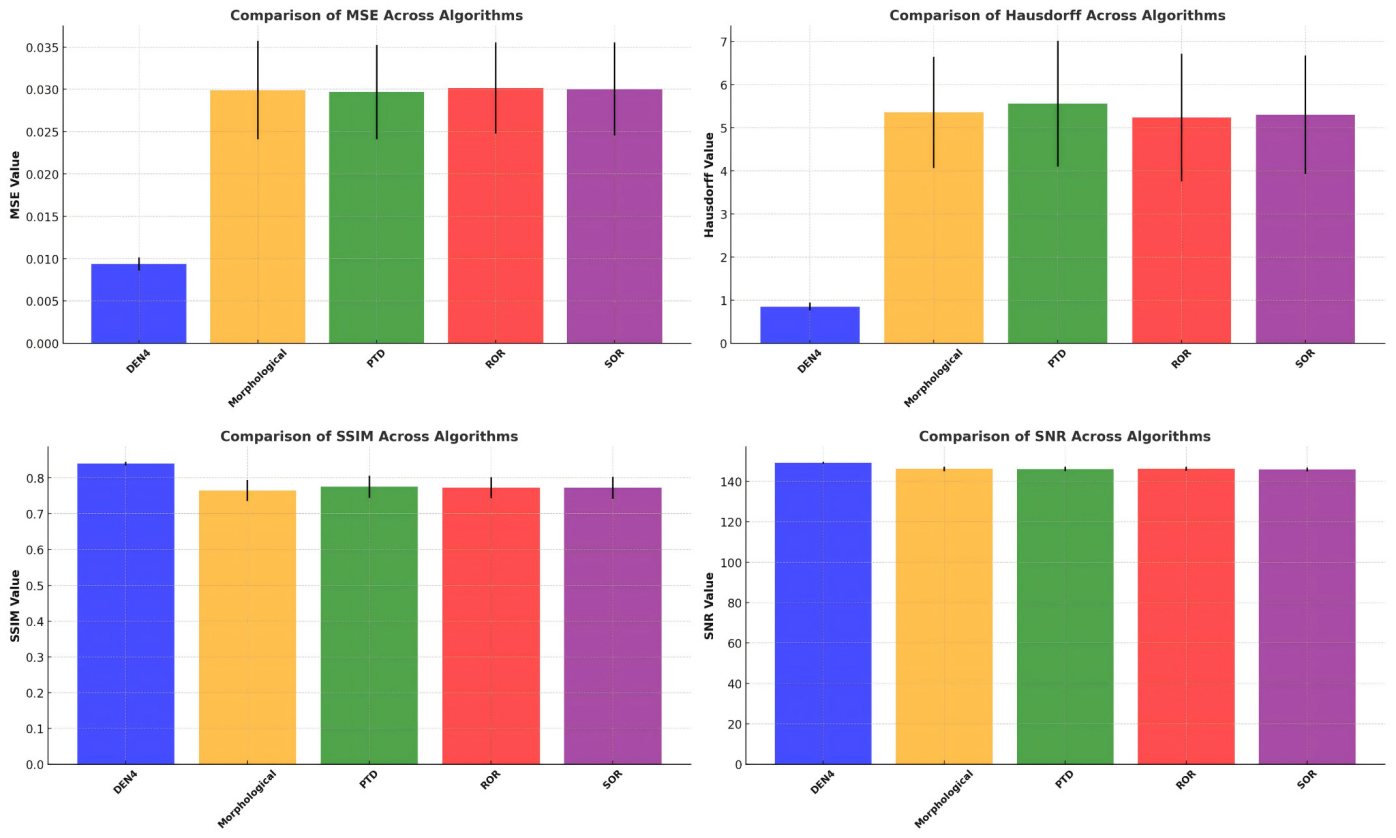
Comparison of S10 before and after denoising.

**Figure 4:**
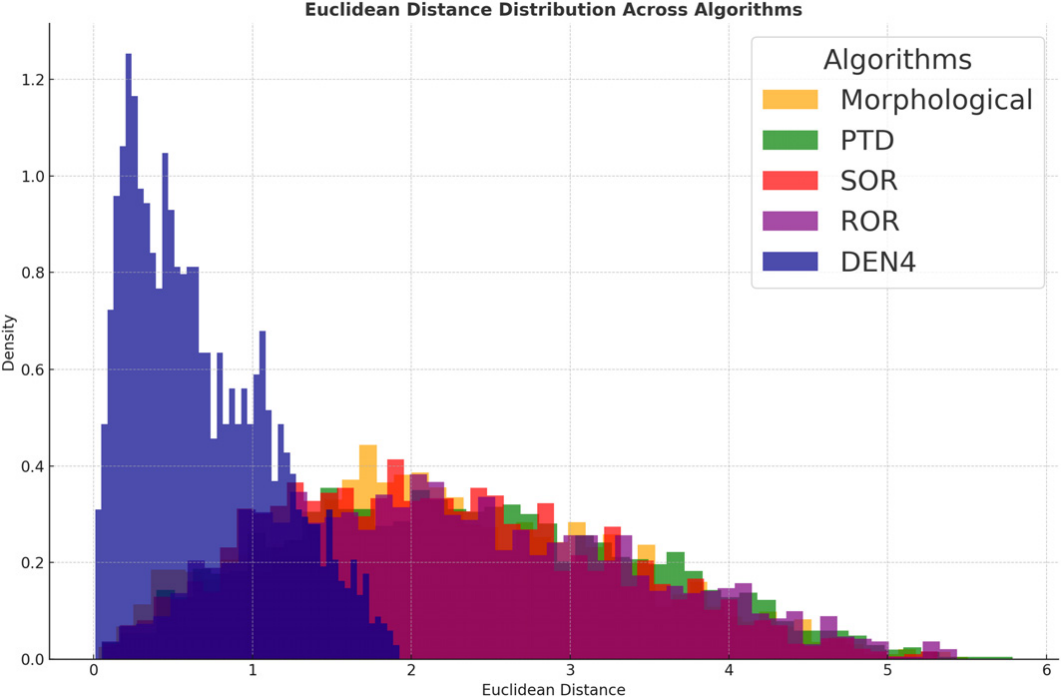
Euclidean distance distribution across algorithms.

This paper employs visualization methods to further present the experimental data. **Figure 3** illustrates the mean and standard deviation of four key metrics—MSE, SNR, Hausdorff distance, and SSIM—for different algorithms. **Figure 4** shows the Euclidean distance distribution of five algorithms: DEN4, Morphological, PTD, SOR, and ROR.

In **Figure 3**, DEN4’s distribution is highly concentrated, mainly within a small distance range (0 to 1), with the highest density peak. This indicates that DEN4 produces more compact denoising results, with smaller inter-point distances, reflecting superior denoising performance and stability. In contrast, the distributions of Morphological, PTD, SOR, and ROR are more dispersed, particularly with higher densities in the large-distance range (2 to 5). This suggests weaker denoising performance, potentially leading to point cloud diffusion or fragmentation.

**Figure 4** reveals that DEN4 achieves the lowest MSE and the smallest standard deviation, demonstrating its significant advantage in minimizing errors compared to other algorithms. Other methods, such as Morphological and PTD, exhibit higher MSE values and greater error variability. DEN4 also achieves a significantly higher SNR than the other algorithms, indicating the best signal quality after denoising. While the SNR values of other algorithms are relatively close, they are consistently lower than DEN4.

Regarding Hausdorff distance, DEN4 achieves the lowest value, highlighting its excellent ability to preserve the geometric structure of the point cloud. Other algorithms, particularly Morphological and PTD, show much higher Hausdorff distances, indicating poorer geometric preservation. Moreover, DEN4 achieves the highest SSIM value with minimal variation, confirming its superior ability to maintain the structural similarity of the point cloud.

#### 3.4.2 Visual evaluation

**Figure 5:**
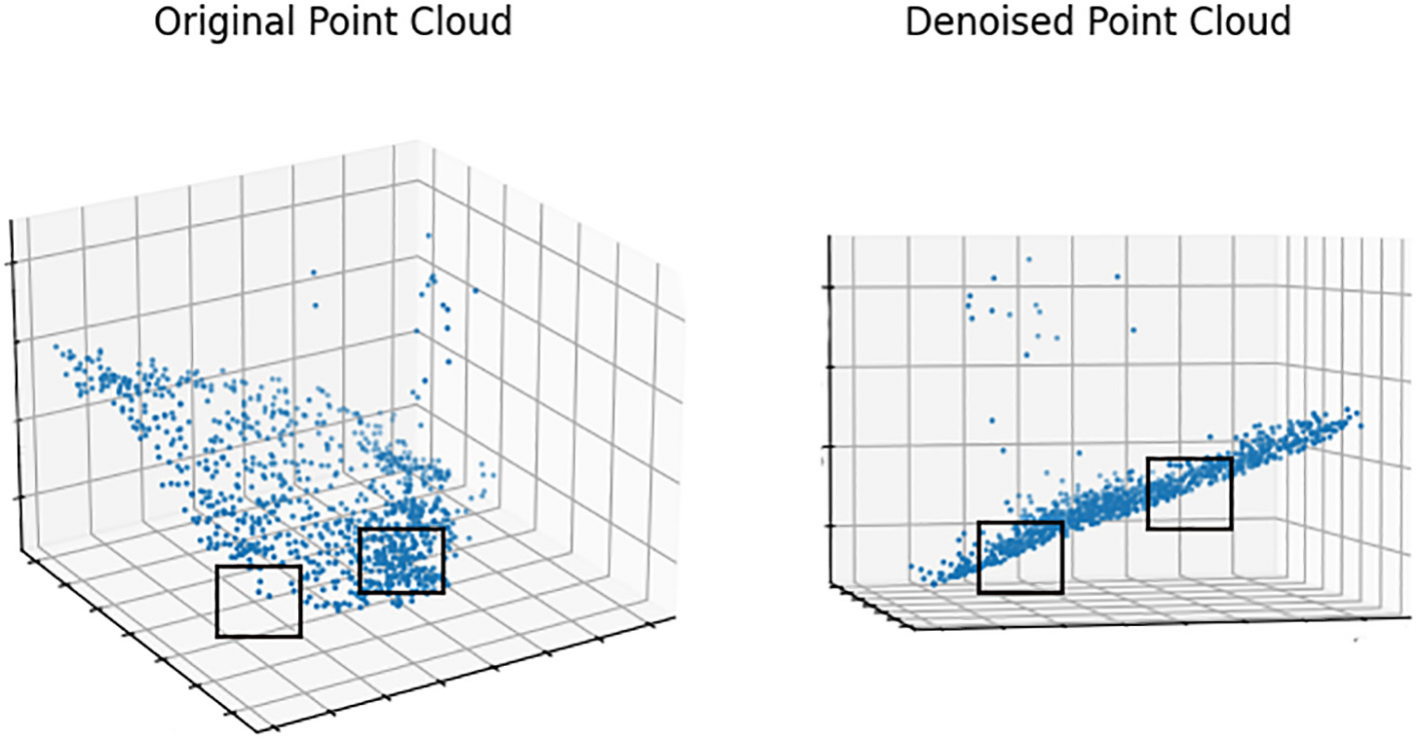
Comparison of S10 before and after denoising.

In **Figure 5**, this paper examines the denoising effect on the S10 point cloud by sampling and displaying 10,000 randomly selected points from the processed data. The results are very clear. In the original point cloud, the areas marked by black boxes exhibit uneven thickness and contain a significant amount of noise. However, in the point cloud processed by the algorithm, the thickness is generally consistent, and the distances between the points have reached a more ideal state. The denoising process significantly improved the overall smoothness of the point cloud, reducing the aggregation of large noisy point clusters and minimizing floating noise points, resulting in a more uniform and clearer point cloud dataset.

**Figure 6:**
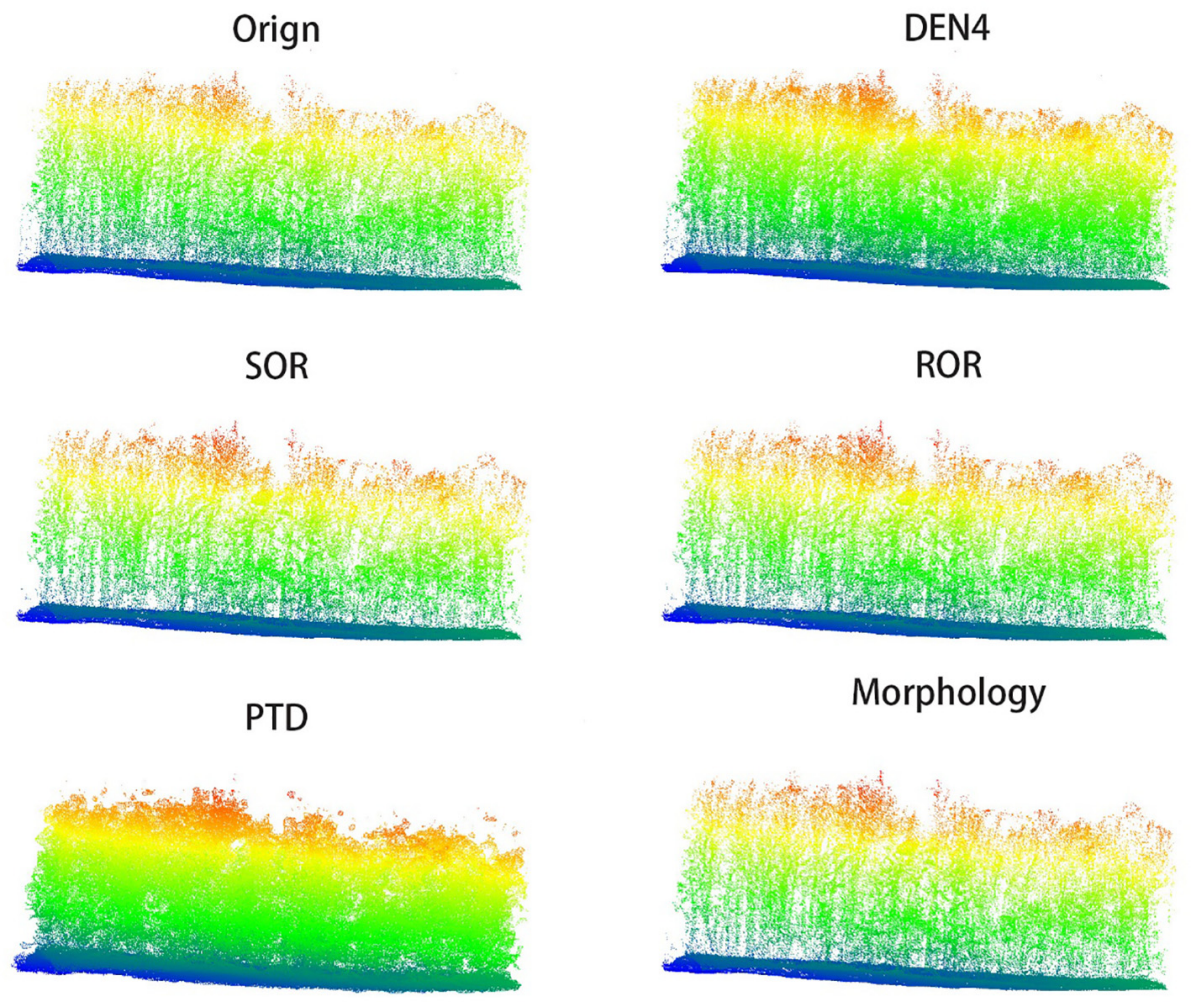
Front view comparison of S5 point cloud.

**Figure 7:**
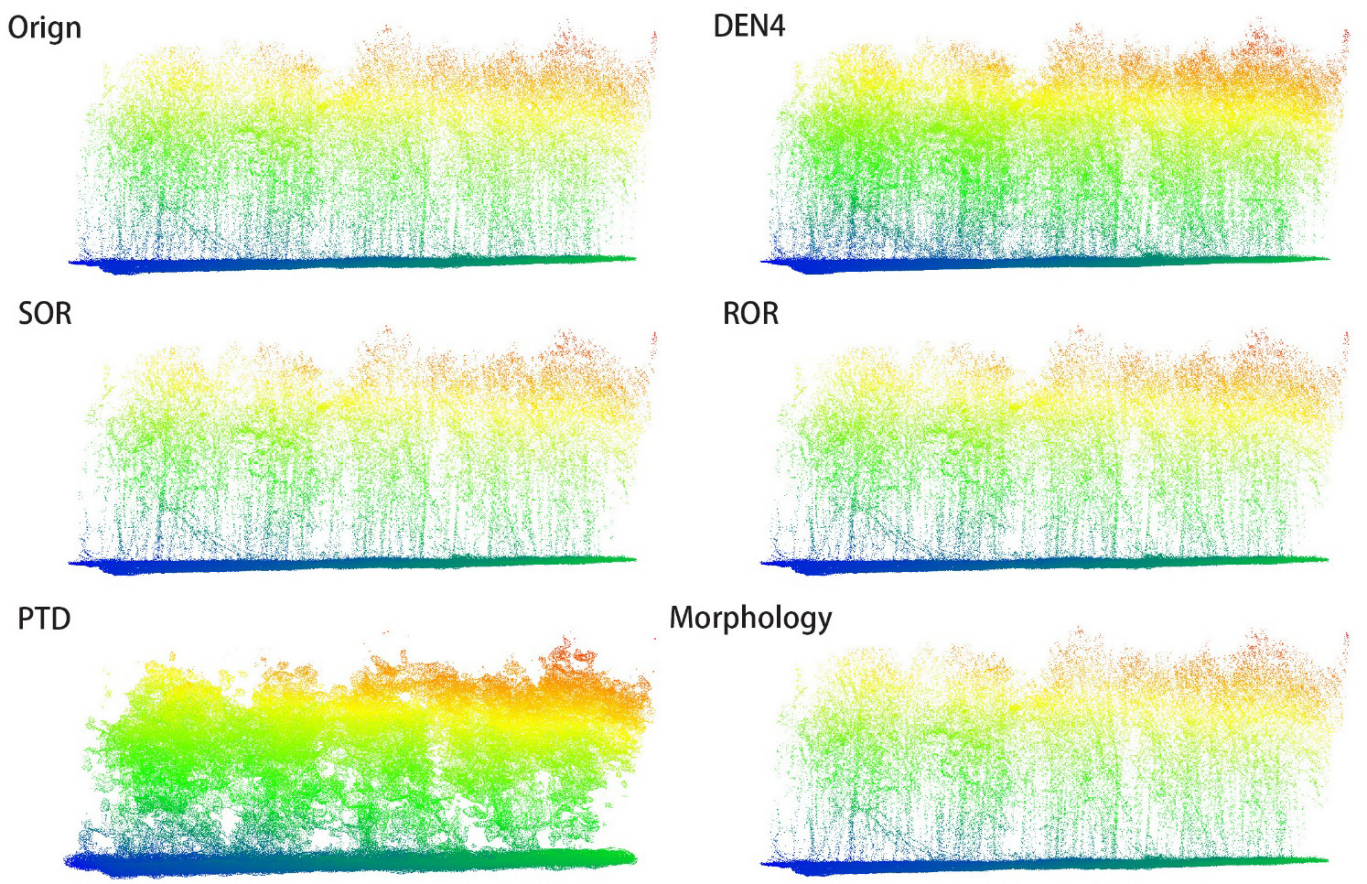
Front view comparison of S10 point cloud.

In **Figures 6**, **7**, we present the results of processing the S5 and S10 point cloud data using different algorithms, alongside the frontal view of the original point cloud. Compared to other methods, our algorithm more accurately captures the contours of individual trees, while maximizing the preservation of critical features such as canopy width, tree height, and diameter at breast height. Specifically, in the upper regions, our algorithm exhibits denser color, indicating that the outer contour areas of the point cloud are more densely populated with fewer noise artifacts, and the point cloud outside the canopy is more concentrated. This highlights the superior performance of our algorithm in canopy recognition, with more complete feature retention. Additionally, the algorithm excels in trunk recognition, clearly delineating the overall outline of the tree trunk. In contrast, other algorithms show deficiencies in analyzing the overall tree structure and contours, leading to the loss and deformation of some details. These traditional methods struggle to adequately preserve the natural contours of trees, particularly in maintaining canopy and trunk features. In comparison, our algorithm better retains the overall structure of the tree, avoiding significant deformation and information loss, resulting in more accurate and realistic processing outcomes.

**Figure 8:**
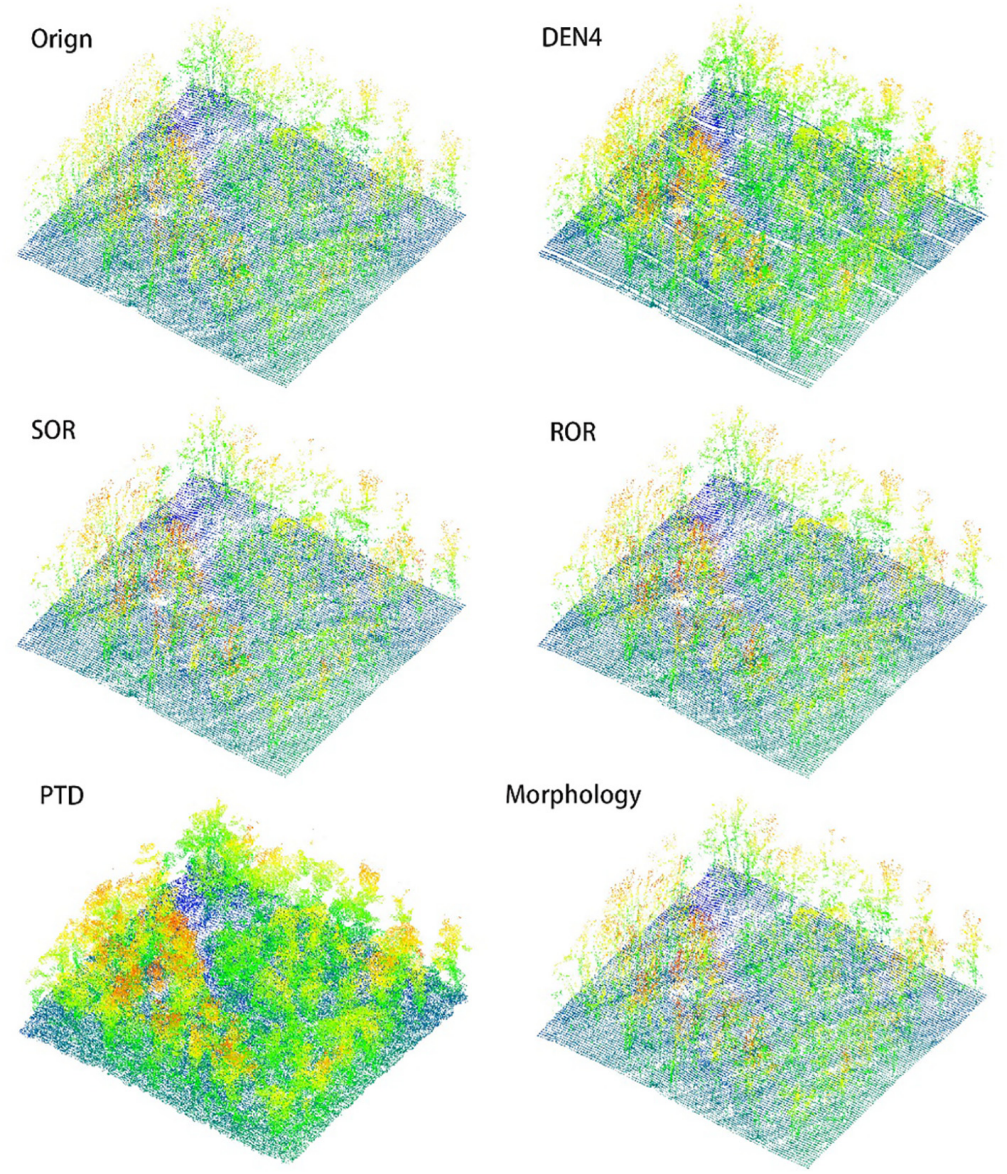
Overhead view comparison of S5 point cloud.

**Figure 9:**
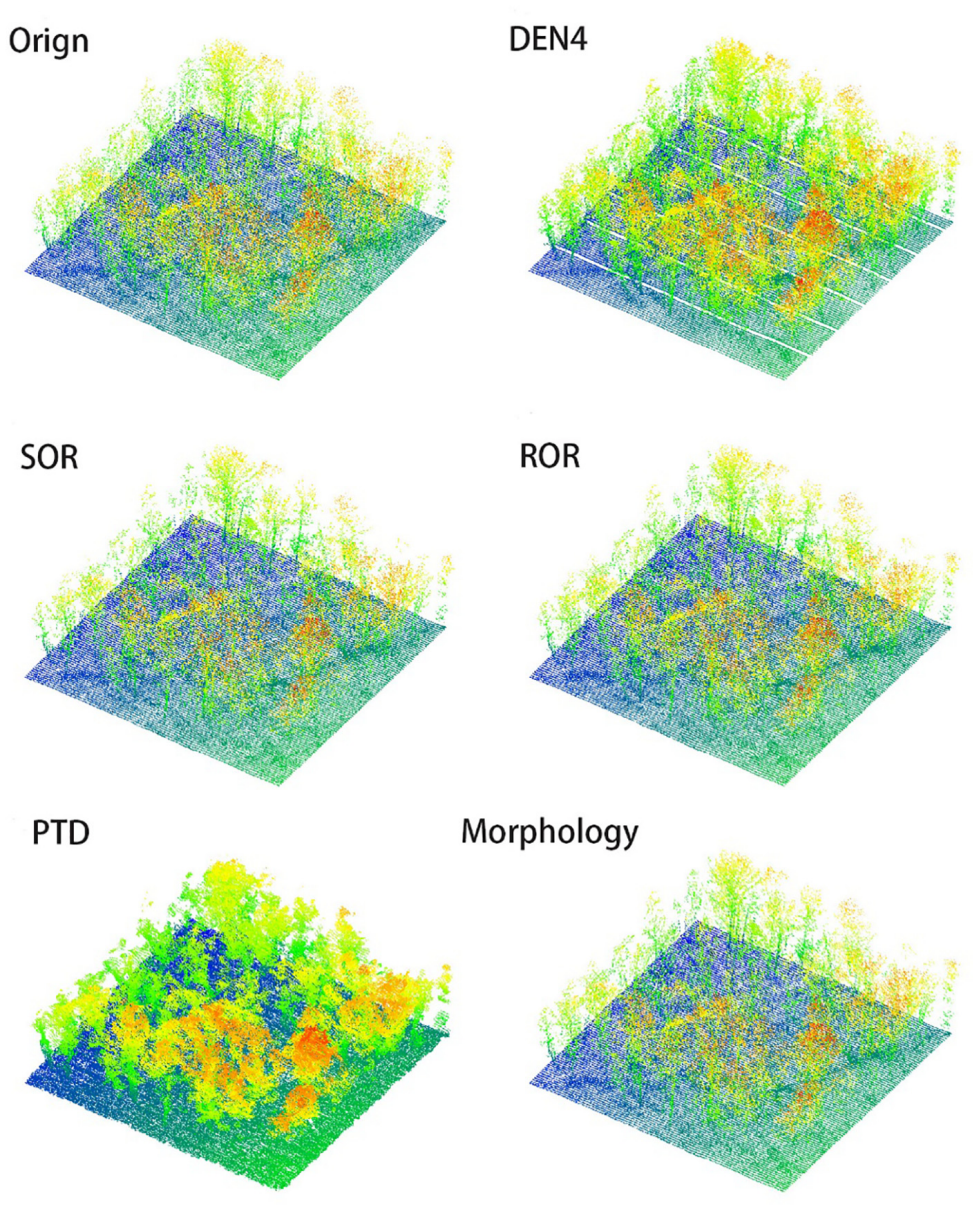
Overhead view comparison of S10 point cloud.

In **Figures 8**, **9**, we present the results of processing the S5 and S10 point cloud data using different algorithms, along with the overhead view of the original point cloud, to compare the overall shape and canopy structure after denoising. The method employed in this study demonstrates exceptional performance in preserving the overall canopy features, effectively removing most of the noise, and rendering the primary shape of the point cloud clearer and more complete. Compared to other algorithms, this method not only better retains the canopy characteristics but also significantly reduces the impact of noise on the point cloud’s shape, thereby enhancing the visual quality of the denoised results.

## 4 Discussion

### 4.1 Results discussed

In this study, we propose an innovative unsupervised deep learning-based point cloud denoising algorithm, DEN4, designed to enhance the denoising performance of single-tree segmentation in LiDAR point clouds. By improving the denoising process, this method aims to facilitate more accurate single-tree segmentation and related research.

DEN4 introduces a multi-level noise separation module into its network architecture, effectively distinguishing between signal and noise. This mechanism ensures the efficient removal of significant noise while preserving the fine structures of the point cloud, significantly reducing errors and improving the signal-to-noise ratio (SNR). The dual noise-handling design makes DEN4 particularly suitable for denoising LiDAR point clouds in complex scenarios, ensuring consistent performance across diverse datasets. Our results demonstrate that DEN4 achieves an MSE mean of 0.0094, substantially lower than Morphological (0.0299) and PTD (0.0297), with a standard deviation of just 0.0008, indicating reduced error and exceptional stability. On the S10 dataset, DEN4 reduced MSE by 70.2% compared to Progressive Triangulated Filtering and by 37.8% compared to Radius Outlier Removal. Additionally, DEN4 achieves an average SNR of 149.1570, significantly outperforming Morphological (146.1743) and PTD (146.1301). Notably, in the S5 dataset, DEN4’s SNR increases to 149.4042, highlighting its superior signal retention capabilities.

Inspired by PointNet and DGCNN architectures, DEN4 refines the local feature extraction module, capturing intricate details of the point cloud with precision. This design ensures the preservation of local geometric details, such as tree height and diameter at breast height, during the denoising process, effectively mitigating common issues in traditional methods, such as detail loss and over-smoothing. As a result, DEN4 maintains the core structural consistency of point clouds across different scales, significantly improving geometric shape retention. Experimental results corroborate this capability: DEN4 achieves an average Hausdorff distance of 0.8503, significantly lower than Morphological (5.3568), PTD (5.5593), and SOR (5.3034). On the S5 dataset, the Hausdorff distance further decreases to 0.8116, demonstrating DEN4’s outstanding ability to preserve complex geometric structures.

By integrating global and local feature learning, DEN4 ensures that the denoising process not only focuses on fine details but also maintains the overall structural consistency of the point cloud. This global feature learning capability effectively prevents the shape distortions commonly observed in traditional filtering methods, ensuring that the denoised point cloud closely aligns with the original data. The unsupervised learning mechanism further enhances the algorithm’s adaptability to various datasets, resulting in consistent performance across diverse environments. This adaptability is reflected in the SSIM metric, where DEN4 achieves an SSIM of 0.8399, outperforming other algorithms in most experiments, with minimal variation (standard deviation of only 0.0054). This demonstrates DEN4’s stable performance in preserving the overall structure of the denoised point cloud.

As an unsupervised algorithm, DEN4 requires no pre-labeled data and can adaptively capture the characteristics of different datasets. This feature enables DEN4 to maintain consistency and robustness across diverse forest point cloud datasets. Particularly in complex data environments, the unsupervised learning mechanism ensures high consistency between the denoised point cloud and the original data, enhancing the algorithm’s generalizability in various scenarios. This robustness is evidenced by DEN4’s MSE standard deviation of just 0.0008, significantly lower than other algorithms, indicating minimal error fluctuation during the denoising process. Similarly, DEN4’s SNR standard deviation of 0.5628 is far lower than Morphological (1.1729) and PTD (1.1254), highlighting its stable signal quality.

This study introduces a comprehensive suite of innovative designs—including dual noise processing, local feature extraction, global feature learning, and unsupervised learning—that collectively elevate the quality and stability of point cloud denoising to a new standard. The effectiveness of these advancements is evident in their superior performance across key metrics: dual noise processing achieves optimized MSE and SNR, local feature extraction significantly reduces Hausdorff distance while preserving geometric structures, global feature learning enhances SSIM stability, and the unsupervised learning mechanism ensures consistent denoising performance across diverse datasets.

The seamless integration of these design elements enables DEN4 to produce exceptionally clean and accurate point clouds while preserving intricate details, offering powerful support for single-tree segmentation and forest resource management applications. These multifaceted improvements not only validate the algorithm’s robustness but also highlight its potential for transformative applications in complex ecological environments.

### 4.2 Conclusion

Lidar point cloud data is an invaluable resource in the field of forest survey and management, providing essential parameters and metrics that are vital for the effective monitoring and management of forest resources. Despite its importance, traditional point cloud processing algorithms frequently encounter limitations pertaining to accuracy, robustness, and the maintenance of contour integrity. These constraints impede the ability to fully exploit the data in complex, real-world settings. In response to these challenges, this study undertook an extensive investigation using a dataset comprising 17 forest plots located in Qingyuan City, Guangdong Province. Following a comprehensive data preprocessing phase, an unsupervised deep learning algorithm, specifically designed for point cloud denoising, was introduced and further refined. The structure of the algorithm was meticulously redesigned to more closely align with the specific requirements of individual tree extraction tasks. By systematically introducing varying levels of noise into the dataset and employing multi-layer deep learning techniques, this study was able to effectively generate cleaner and more accurate point cloud data. The results highlight the potential of the proposed algorithm to enhance the precision and reliability of point cloud data, thereby contributing to more effective forest resource monitoring and management.

The experimental results demonstrate that the improved algorithm not only adapts effectively to various individual tree extraction Lidar point cloud datasets but also significantly enhances the denoising process, resulting in clearer tree outlines. The algorithm excels in preserving crucial features such as tree height and diameter at breast height, ensuring that these key characteristics remain intact. Compared to traditional algorithms, this method shows remarkable superiority in both quantitative analysis and visual results, highlighting its robustness and precision in maintaining the structural integrity of the point cloud data. The clear improvement in noise reduction and feature preservation underscores the algorithm’s potential for widespread application in the domain of individual tree extraction using Lidar point cloud denoising. Its ability to deliver consistent and reliable results across different datasets further validates its applicability in practical scenarios, making it a promising tool for enhancing the accuracy and effectiveness of forest resource monitoring and management.

Although this study demonstrates the superiority of the algorithm in denoising Lidar point clouds for individual tree extraction, there is still room for improvement. Since the dataset used primarily consists of plots with a single tree species, the algorithm’s effectiveness in processing other single-species and multi-species scenarios has yet to be validated. It is recommended to further explore the algorithm’s potential applications in complex mixed forests or large-scale forest surveys. Special attention should be given to investigating the impact of different seasons and environmental factors (such as light, humidity, and vegetation density) on the algorithm’s performance to further enhance its generality and robustness. Therefore, future research should aim to diversify the dataset to test the algorithm’s applicability across different tree species and complex forest environments. Additionally, future studies should focus on developing a comprehensive deep learning algorithm for individual tree extraction and integrating it with other optimized algorithm models.

This approach will be instrumental in better addressing the challenges encountered in practical applications, particularly when handling large-scale forest datasets. The complexity of real-world environments, including the diversity of forest types and the varying conditions under which data is collected, requires algorithms that are not only robust and adaptive but also capable of delivering precise results across different contexts. By integrating the strengths of multiple algorithms, future research can aim to develop point cloud processing methods that are both more efficient and more accurate. Such methods will be crucial in meeting the increasingly sophisticated demands of forest resource surveying and management, where the need for high-resolution, reliable data is ever-growing. The development of these advanced methodologies will not only enhance the accuracy of individual tree extraction and related tasks but will also contribute to the broader field of environmental monitoring and conservation by providing tools that are capable of coping with the complexities of natural ecosystems on a large scale.

## Data Availability

The original contributions presented in the study are included in the article/supplementary material. Further inquiries can be directed to the corresponding author.
